# Pilot study of dogs with suppurative and non-suppurative *Malassezia* otitis: A case series

**DOI:** 10.1186/s12917-021-03066-7

**Published:** 2021-11-18

**Authors:** Tania C. Nunes Rodrigues, Sophie I. Vandenabeele

**Affiliations:** Small Animal Department, Faculty of Veterinary Medicine, Ghent University, Salisburylaan 133, 9820 Merelbeke, Belgium

**Keywords:** *Malassezia*, hypersensitivity, suppurative, otitis, inflammation, canine, histopathology

## Abstract

**Background:**

Rarely, *Malassezia* otitis presents as a painful, erosive otitis with an otic discharge containing *Malassezia* and neutrophils on cytology. There are no published reports of this type of suppurative *Malassezia* otitis (SMO). The role of *Malassezia* hypersensitivity in otitis is still unknown, and no association has been demonstrated with SMO. We compared *Malassezia* IgE levels, intradermal test and histology changes in SMO dogs with the more conventional *Malassezia* otitis (MO) presentation.

**Results:**

Three dogs (case 1, case 2 and case 3) were diagnosed with SMO, one dog (case 4) was diagnosed with unilateral MO and unilateral SMO, and one dog (case 5) was diagnosed with MO. Only one case (case 4) with SMO/MO had a positive Intradermal Allergy Test (IDAT) and elevated IgE levels for *Malassezia*. Histopathology findings from SMO revealed: interface dermatitis (case 1 and 3), lymphocytic dermatitis (case 2) and chronic hyperplastic eosinophilic and lymphoplasmacytic dermatitis (case 4). Histopathology findings from MO showed perivascular dermatitis (case 4 and 5). All the cases were treated successfully.

**Conclusions:**

SMO presents with a distinct clinical phenotype in comparison with conventional MO. No consistent aetiology could be isolated. In these clinical cases it is possible that previous treatments could have influenced the results. More research is needed to understand the possible aetiologies and the pathogenesis of SMO.

## Background

*Malassezia pachydermatis* is commonly involved in canine otitis externa [1, 2]. Changes in the microenvironment and defective host immunity seem to be associated with *Malassezia* overgrowth [3]. Contact, immediate or delayed hypersensitivity to *Malassezia* has been reported in dogs [3]. Furthermore, clinically healthy dogs have not been shown to elicit an immediate hypersensitivity response to intradermal injection of *M. pachydermatis* extracts, in contrast, dogs with *Malassezia* otitis do [4]. This supports the possibility that a type I hypersensitivity response could be important in Malassezia otitis's pathogenesis and might indicate the need for a rigorous antimycotic treatment [3] or allergen-specific immunotherapy (ASIT) [5]. *Malassezia pachydermatis* can also be associated with biofilm production and decreased antifungal susceptibility [6, 7].

*Malassezia* otitis can present as overgrowth alone or with inflammatory cells in a concurrent exudative bacterial otitis [8]. The authors have recognised another type of chronic *Malassezia* otitis that presents with pain, a suppurative exudate, and sometimes with ulceration of the ear canal. Cytology is the only test to differentiate between the more conventional *Malassezia* otitis (MO) and suppurative *Malassezia* otitis (SMO), in which yeast (in the absence of bacteria and other obvious inflammatory stimuli) is present with inflammatory cells [9].

We postulated that SMO could be associated with more severe histopathological abnormalities in the ear canal, and sensitization to *Malassezia* could be more frequent compared with classic MO.

## Results

**Fig. 1:**
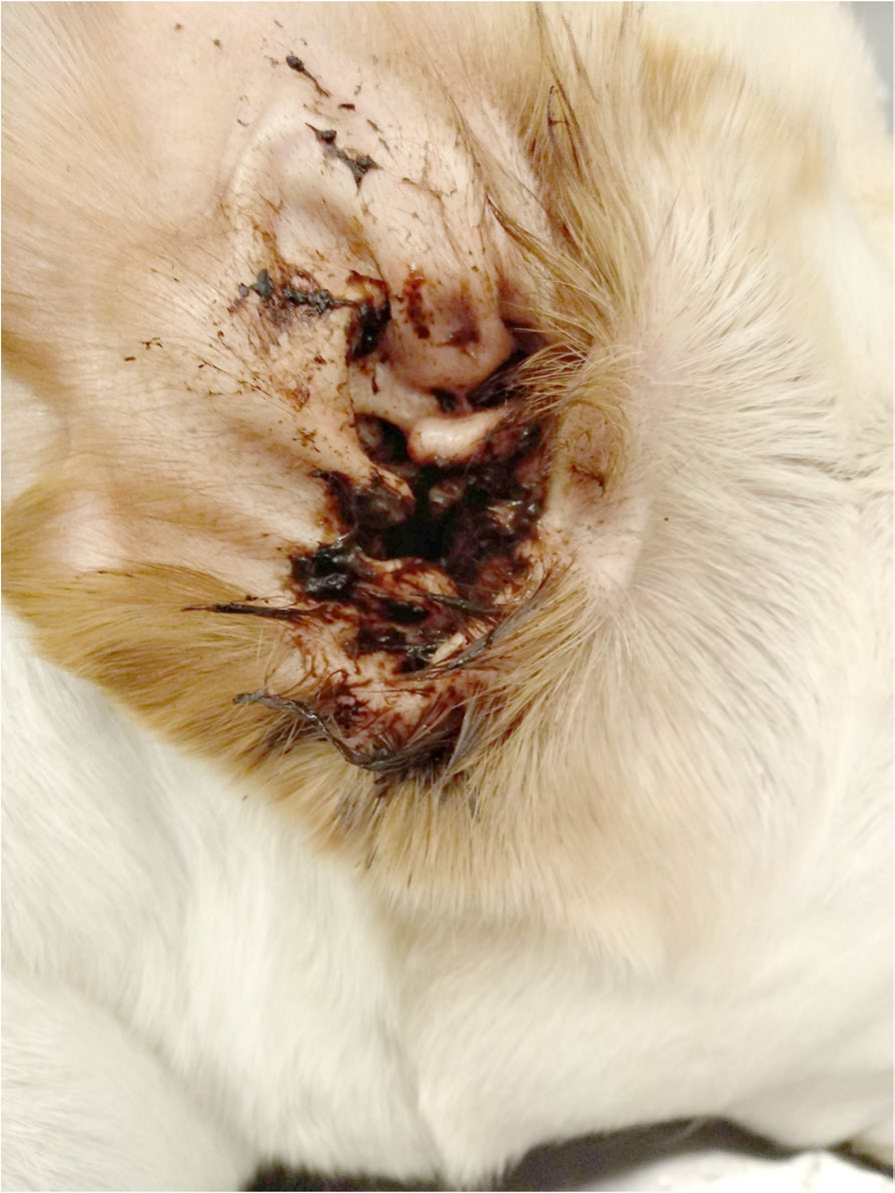
Right ear with dark watery discharge on the pinna and entrance of ear canal (case 1)

The affected ears with SMO presented with brown to dark watery exudate (Fig. 1) whereas the ears affected with classical MO showed a ceruminous discharge. No other body sites were affected in these cases except for case 4 that had a history of licking the paws without associated infection.

The video-otoscope examination of each ear canal (Table 1) revealed that case 1 (bilateral SMO) both ears presented with OTIS3 of 10. Case 3 (bilateral SMO), the left ear had a slightly lower OTIS3 (OTIS3=9) than right ear (OTIS3=10). Case 2 (unilateral SMO) and SMO of Case 4 showed the same value of OTIS3 (OTIS3=9). The ear canals with MO had a lower OTIS3 value (Case 4 OTIS3=3; Case 5 OTIS3=6). The normal ear canals of the case 2 and case 5 showed a OTIS score of 0.

All ear canals of the 5 cases had an intact tympanic membrane (TM) however the TM was opaque in the affected ear canals.

**Table 1:** Otitis Index Score results for each ear canal

OTIS3	Erythema		Edema/swelling		Erosion/ulceration		Exudate		Total	
Ear	Left ear	Right ear	Left ear	Right ear	Left ear	Right ear	Left ear	Right ear	Left ear	Rightear
**Case 1**	3	3	2	2	2	2	3	3	10	10
**Case 2**	0	2	0	2	0	2	0	3	0	9
**Case 3**	2	2	2	2	2	3	3	3	9	10
**Case 4**	1	2	1	2	0	2	1	3	3	9
**Case 5**	2	0	1	0	0	0	3	0	6	0

**Fig. 2:**
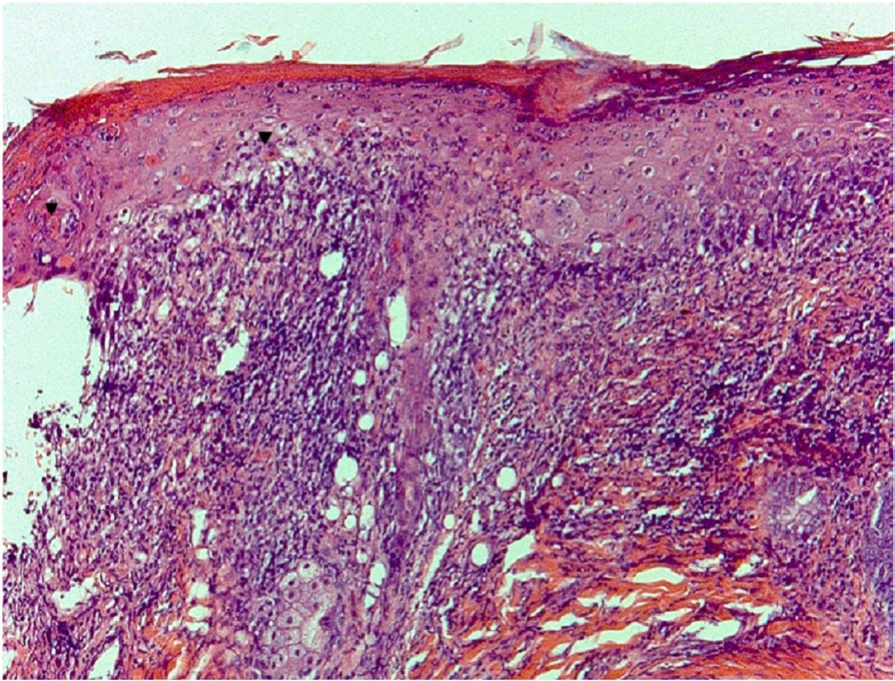
Haematoxylin and eosin photomicrograph (×10 magnification) of affected area of the entrance of ear canal (case 1) revealing apoptotic keratinocytes surrounded by lymphocytes (satellitosis) (black arrowheads). Note vacuolar degeneration of the basal layer and dermis with band-shaped of inflammatory infiltrates

Only case 4 had a positive result for IDAT and for IgE serology. The IDAT results and histopathology findings are described in Table 2. Histology of case 1 is documented in Fig. 2.

**Table 2:** Allergy tests and histopathology results

	Control Positive	Control Negative	IDAT Malassezia at 15’ min	IDAT Malassezia at 30’min	Serology IgE (EAU)	Histopathology
Case 1	4+	0	0	0	Not performed	Left and right ear SMO: Lichenoid- interface dermatitis with apoptosis
Case 2	4+	0	0	0	145 (reaction class 0)	Right ear SMO: Minor chronic lymphocytic dermatitis
Case 3	4+	0	0	0	91 (reaction class 0)	Left and right ear SMO: Interface dermatitis with rare basal degeneration
Case 4	4+	0	3+	2+	3090 (reaction class 5)	Left ear MO: Perivascular dermatitis Right ear SMO: chronic hyperplastic eosinophilic and lymphoplasmacytic dermatitis
Case 5	4+	0	0	0	0 (reaction class 0)	Left ear MO: Lymphocytic dermatitis and focal detachment of the epidermis

Abbreviations: *EAU* ELISA absorbance units. Interpretation EAU: reaction class 0 no antibodies present [0-150 EAU], reaction class 1 equivocal [151-300 EAU], reaction class 2 low concentration of antibodies [301-600 EAU], reaction class 3 moderate concentration of antibodies [601-1200EAU], reaction class 4 high concentration of antibodies [1201-2400 EAU], reaction class 5 very high concentration of antibodies [>2400 EAU].

All cases have the first follow-up after four weeks of starting treatment, except case 4. Case 1 was treated with alternate days of prednisolone at 0.5 mg/kg, oral ketoconazole at the dose of 5mg/kg daily and topical orbifloxacin, mometasone furoate monohydrate and posaconazole (Posatex®, Intervet; Boxmeer, the Netherlands) once a day into each ear. At the follow-up, otitis was resolved and treatment was stopped. Case 2 was discharged with oral prednisolone at 0.8 mg/kg q24h for 14 days then alternate days, oral ketoconazole 5 mg/kg daily, and topical Posatex® on affected ear q24h. At follow-up, there was Malassezia 1+ on the affected ear therefore, Posatex® was continued, ketoconazole stopped and the dose of prednisolone decreased to 0.4 mg/kg on alternate days until the second follow-up in 4 weeks. At that time, SMO was resolved and medication was stopped. Case 3 was treated with oral prednisolone at 0.4 mg/kg q24h for 14 days, then alternate days for 14 days. Topical Posatex® into each ear once a day and TrizEDTA® ear cleaner (Dermapet, US) three times a week. After one month, the left ear was normal and the right ear showed Malassezia yeast 1+, the prednisolone was reduced to 0.25 mg/kg on alternate days, Posatex® was continued daily and ear cleaner discontinued. Two months after start treatment, otitis was resolved and treatment stopped. Case 4 was treated with oral prednisolone at 0.5 mg/kg q24h for 14 days, then alternate days for 14 days. Oral ketoconazole was prescribed 5 mg/kg daily, topical Posatex® once a day into each ear and TrizEDTA® ear cleaner once a week. The patient came for a revisit after three months and otitis in both ears was resolved. The dog was on pulse therapy of ketoconazole after four weeks daily, 0.25 mg/kg on alternate days of prednisolone and twice a week Posatex®. The medication was discontinued and a strict food trial with novel protein was suggested due to a history of pedal pruritus. However, the diet trial was stopped due to dog/client compliance few weeks later. The treatment for case 5 comprised oral prednisolone at 0.5 mg/kg q24h for 14 days, then alternate days for 14 days. In addition, topical Posatex® into affected ear daily and TrizEDTA® ear cleaner three times a week. At the follow-up, MO was resolved and medication discontinued. The cases revealed no further relapse twelve months post-presentation at our facilities, except case 1 was euthanised due to unrelated disorders.

## Discussion

To the authors' knowledge, this is the first time that SMO has been reported.

Clinically SMO presented with aural purulent discharge associated with pain. This is clinically different from the ceruminous exudate and pruritus commonly related to MO [3]. SMO cases had a OTIS3 value higher than MO cases.

SMO seems to be associated with a more chronic presentation (3 months to 5 years), and therefore it is unclear if previously administered topical therapies play a role in this type of *Malassezia* otitis. In this case series, at least two cases did not receive any topical treatment at the time of presentation.

Cytologically, SMO presented with neutrophils and might resemble a *Malassezia* biofilm described by Paterson [10]. However, the evidence supporting this cytology's correlation using Diff Quik stains with biofilm formation is limited. *Malassezia* biofilm formation has only been demonstrated in vitro [6, 7]. Therefore, we can't be sure of the involvement of an actual biofilm in SMO cases or the role of neutrophils with *Malassezia* biofilm. Other organisms could have been present in the ear canal responsible for neutrophils but not detected on cytology like rod shape bacteria (e.g. *Pseudomonas spp.*) or cocci. This might explain the neutrophils' presence in one ear and not in the other ear in the same dog (case 4). In reflection, bacterial culture and susceptibility testing could have been beneficial for a better understanding of SMO. Another type of *Malassezia species* might be involved in SMO cases. In human medicine, malignant otitis externa (MOE) usually affects immunosuppressive individuals and is usually associated with Pseudomonas aeruginosa [11]. However, fungal pathogens have also been associated with MOE and *Malassezia sympodialis* was reported as a cause of MOE [12]. Pain and purulent aural discharge that starts in the external auditory canal and its extension to the temporal bone can result in osteomyelitis and consequent cranial nerve palsies and intracranial infection [11, 13]. Furthermore, otitis media is an important cause of the recurrence of otitis externa, with bacterial infection responsible for most infections. However, yeast has also been associated with middle ear disease and identified in 23.7% of cases with otitis media [14]. More recently, *Malassezia* otitis media was diagnosed in 17% of cases of chronic *Malassezia* otitis by otoscopy and sampling of middle ear content in 14 cases [15]. There was evidence of soft tissue attenuation material in the tympanic bulla on CT imaging in 4 of those cases [15]. In reflection, myringotomy, imaging, fungal culture and microbiome studies could have brought more information about lesion extension and the mycobiota of the SMO.

We postulated that SMO could be associated with sensitization to *Malassezia* when compared with the classic MO. However, case 4 with unilateral conventional MO, unilateral SMO and pedal pruritus was the only one with IDAT and *Malassezia*- specific IgE results consistent with *Malassezia* hypersensitivity. This was also the case with non-infectious pedal pruritus. Hence in that patient, other clinical complaints were pointing towards allergic dermatitis. All the remaining SMO cases had negative results for the allergen-specific IgE and IDAT for *Malassezia*. This outcome is contrary to that of Kim and colleagues, who stated dogs with *Malassezia* otitis would manifest immediate hypersensitivity response in the IDAT [4] though the possibility of prednisolone suppressing test results in case 1 and 2 need to be noted. The authors recognise the importance of withdrawing times to assess immediate reactions to IDAT and allergen-specific IgE serological testing as recommended by Olivry and Saridomichelakis [16]. This fact was particularly important for case 1 and 2 that were on prednisolone at presentation but showed a positive control but both IDAT and IgE serology were negative (Case 2). Case 2 might have been hypersensitive to *Malassezia* but suppressed by long treatment with prednisolone.

Atopic dogs have also been reported to have significantly higher IgE antibodies to *Malassezia* than non-affected dogs or non-atopic dogs with *Malassezia* dermatitis/otitis [17]. However, the potential for underlying causes as atopic dermatitis was not investigated during this study. Up to the present moment, none of the dogs with SMO in this cases series relapsed. Therefore, atopy underlying to SMO seems unlikely in these cases except for case 4. The data from this study, albeit limited, included the confounding finding in the only dog with significant IgE reactivity to *Malassezia* showing SMO in one ear and conventional MO in the other, which does not support immediate hypersensitivity to *Malassezia* as the primary cause for SMO.

Histopathology findings of the affected ear canals showed variable results. This might be due to the selection of biopsy site, duration of disease and type of topical medication used. In case 1 (SMO), the histopathological result of lichenoid-interface dermatitis suggested the possibility of a drug reaction to previous treatment [18]. This case was treated topically upon one week before the presentation with a topical magistral solution containing enrofloxacin and the histopathological findings were present while the dog was on alternate days of prednisolone. Infection triggered erythema multiforme [18] was also considered but *Malassezia* has never been described as a cause. The histopathology pattern of case 2 (SMO) and case 5 (MO) revealed lymphocytic infiltrate commonly seen in *Malassezia* dermatitis [19]. In case 5, a focal detachment of the epidermis was evident compatible with a possible artefact due to the sampling technique. Case 4, the biopsy of the ear affected by SMO resembled a chronic allergic process (eosinophilic and lymphoplasmacytic infiltrate) and the MO case revealed perivascular dermatitis. Interestingly there was not much difference between MO histopathology (case 4 and 5) and mild changes for SMO (case 2). In case 3, the histopathological pattern had features consistent with a possible of drug reaction (interface dermatitis with rare basal degeneration). However, no medication had been given for four weeks before the presentation, so a drug reaction seems unlikely for this case. The lack of consistency in histopathological changes may reflect distinct pathomechanisms and underlying aetiologies.

The current literature for treatment of MO lacks number for robust clinical trials and a further study with more focus on topical treatment with orbifloxacin, mometasone furoate monohydrate and posaconazole is necessary in MO and SMO cases. All the cases were ultimately treated successfully and the authors considered oral glucocorticoids necessary for the reduction of inflammation and the associated pain of the ear canal during the treatment of SMO.

SMO has been diagnosed, on average, four times a year at the parent institution. The scope of this study was therefore limited in terms of sample size. This study has other limitations, as all the cases were investigated at the day of consultation due to severity of clinical complaints. Because of the SMO patients' aural pain, it was deemed unethical to withdraw all medication for several weeks prior to allergy tests and biopsy sampling. Previous treatments and individual immune response could have altered the results.

## Conclusion

To the best of the authors' knowledge, this is the first description and successful treatment of SMO, a type of otitis with a clinically distinct presentation. Further research should be undertaken to investigate the aetiologies and pathogenesis of SMO.

## Methods

Five unrelated privately owned dogs presented for referral dermatology consultation between May 2019 and March 2020 with complaints of chronic painful otitis externa that did not respond to therapy. For each case a physical and dermatological examination were performed however otoscopy was only possible under sedation due to aural pain. The diagnosis was achieved based on anamnesis, clinical signs (exudate present in the ear canal) and yeast presence (an average of more than 3 yeasts per oil immersion field) with or without inflammatory cells on cytology. It was designated MO for otitis with *Malassezia* yeasts on cytology and SMO for otitis with *Malassezia* yeasts with neutrophils. The clinical data from the five dogs are summarised in Table 3.

**Table 3:** Clinical data of 5 cases diagnosed with *Malassezia* otitis with and without neutrophils

	Breed	Age (years)	Sex	Type of otitis	Duration of otitis	Previous treatments	Therapy at presentation
Case 1	Labrador Retriever	3.1	FS	Bilateral SMO	3 months	Topical Aurizon® Topical Osurnia® Topical acetylcysteine Topical magistral solution containing enrofloxacin	Prednisolone 0.5mg/kg q48h for 5 weeks No topicals treatments for one week
Case 2	Rottweiler	4.3	M	Unilateral SMO	1 year	Ear flush	Prednisolone 0.25 mg/kg q48h for 6 months Ketoconazole 10 mg/kg q24h for 6 weeks
Case 3	Labrador Retriever	10.5	FS	Bilateral SMO	5 years	Osurnia® and Surosolve^TM^	None for 4 weeks
Case 4	Cross Breed	2.9	M	Bilateral: MO on the left ear and SMO on the right ear	2 years	Surolan® Easotic® Ear flush	Meloxicam oral No topical treatments for two weeks
Case 5	Labrador Retriever	4.9	M	Unilateral: MO	14 months	Marbodex® Marbofloxacin PO Surolan®	Ketoconazole 12 mg/kg PO q48h for several months No topical treatments for several months

Ingredients of medication: Aurizon® (Clotrimazole, Dexamethasone, Marbofloxacin), Acetylcysteine (Lysomucil 10%; Zambon S.A., Brussels, Belgium), Surosolve^TM^ (tromethamine-ethylenediaminetetraacetic acid (Tris-EDTA), chloroxylenol, salicylic acid); Marbodex® (marbofloxacin, clotrimazole, dexamethasone), Osurnia® (florfenicol, terbinafine and betamethasone.); Easotic® (hydrocortisone aceponate, miconazole and gentamicin); polymyxin, prednisolone and miconazole (Surolan®; Janssen Pharmaceutica NV, Beerse, Belgium)

Abbreviations: *FS* Female Spayed, *MO* Malassezia otitis, *M* Male, *PO* Per os *SMO* Suppurative Malassezia Otitis

**Fig. 3:**
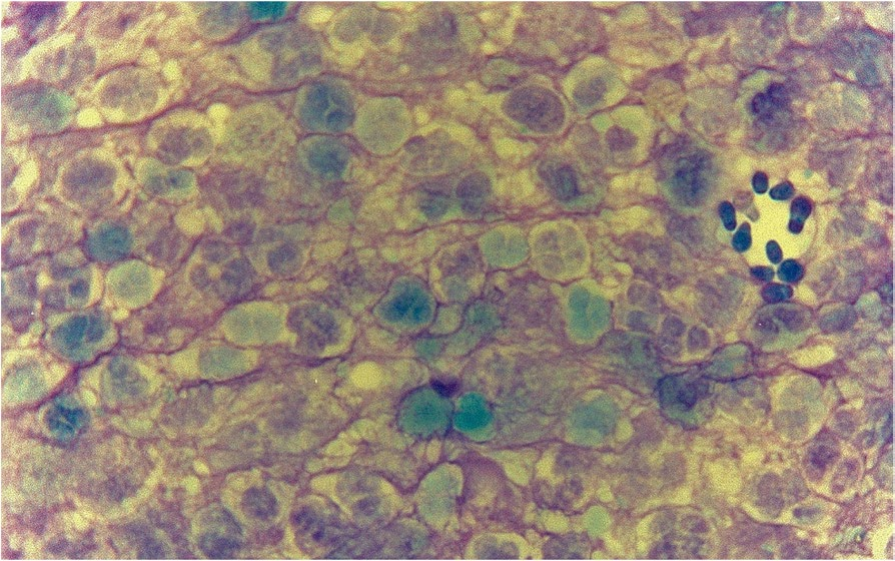
Cytology of left ear smear of case 1 showing *Malassezia* yeasts with neutrophils. Note the purple lace-like filamentous pattern surrounding neutrophils and *Malassezia* yeasts (Diff-Quik, ×100 magnification).

A cotton-tipped swab was inserted into each ear at the vertical and horizontal canal junction to collect exudate. Each sample was rolled onto a glass slide and stained with modified Diff-Quick. The cytology was assessed by microscopic examination under oil immersion. Each slide was scanned on low magnification (40X) to find a representative area for assessment. Once a site was selected, oil immersion was used on 100X magnification to quantify the organisms and presence of inflammatory cells. Another nine adjacent oil immersion fields (OIFs) was scanned for the same purpose. The cytology result for each ear was reported based on the presence of bacteria, yeast and inflammatory cells by semi-quantitative methods [20]. Case 1 and 3 had bilateral otitis externa with 4+ *Malassezia* yeasts with 4+ neutrophils. Case 2 had unilateral otitis externa with 3+ *Malassezia* yeasts with 3+ neutrophils. Case 4 had bilateral otitis externa where the right ear had 2+ *Malassezia* yeasts with 2+ neutrophils and the left ear 4+ *Malassezia* yeasts without neutrophils. The cytology of the SMO cases revealed a lace-like filamentous pattern that obscured *Malassezia* yeasts and neutrophils (Fig. 3). Case 5 had unilateral otitis externa with 2+ *Malassezia* yeasts.

The animals were then enrolled in a prospective study on the day of diagnosis, including sedation for intradermal testing, serology for IgE, ear flush, video otoscopy and biopsy of ear canal.

The animals were sedated with dexmedetomidine (Dexdomitor; Zoetis) 5μg/kg intravenously. The lateral thorax was clipped for intradermal injection of a positive control histamine with a dilution of 1:100 000, negative control (phosphate-buffered saline solution) and *M. pachydermatis* extracts (Stallergenes Greer; USA) with the dilution of 1000 PNU/ml. After 15- and 30-minutes post-injection, the reactions were graded and considered clinically relevant or positive if the subjective wheal and flare reaction were greater or equal than 2+ [21].

A serum sample was sent to an external laboratory (Idexx Laboratories; Hoofddorp, Netherlands) for enzyme-linked immunosorbant assays using the Allergy Test *Malassezia* IgE (GREER; USA) with cross-reactive carbohydrate determinants inhibitor. This was not performed in case 1.

The sedation was converted to general anaesthesia with intravenous methadone (Insistor; Ecuphar) 0.2 mg/kg and intravenous propofol (Propovet; Zoetis) was used to effect. All dogs were intubated and anaesthesia was maintained with isoflurane and oxygen. Prior to the ear flush of the ears, the video otoscope was inserted for examination in each affected ear and the 0-3 Otitis Index Scores 3 (OTIS3) determined (Table 2). The OTIS3 score resulted from the sum of the scores for erythema, edema/swelling, erosion/ulceration and exudate, each parameter was graded between 0 and 3 (total range 0 to 12 )[22].

The affected ear canals were then flushed with warmed sterile saline through a 5 french gauge catheter (Karl Storz; Tuttlingen, Germany) by the working channel of video-otoscope. The integrity of the tympanic membrane of each ear canal was assessed after the complete removal of aural exudate. The affected ear canal was biopsied with a 4-millimetre punch biopsy. Sampling areas were selected on the margin of normal tissue with ulcerative/erosive lesions or an erythematous area depending on the clinical presentation and sent to an external laboratory (Algemeen Medisch Laboratorium, Antwerp, Belgium) for processing and histopathological interpretation. This technique only allowed samples biopsied from the first two centimetres of the vertical portion of the ear canal and no suture material was necessary.

## Data Availability

Data sharing is not applicable to this article as no datasets were generated or analysed during the current study.

## Abbreviations

ASIT: Allergen-specific immunotherapy
IDAT: Intradermal allergy testing
MO: *Malassezia* otitis
MOE: Malignant otitis externa
OTIS3: Otitis index scores 3
SMO: Suppurative *Malassezia* otitis
TM: Tympanic membrane;
